# Hyperimmune Targeting Staphylococcal Toxins Effectively Protect Against USA 300 MRSA Infection in Mouse Bacteremia and Pneumonia Models

**DOI:** 10.3389/fimmu.2022.893921

**Published:** 2022-05-17

**Authors:** Xiaobing Han, Roger Ortines, Ipsita Mukherjee, Tulasikumari Kanipakala, Thomas Kort, Shardulendra P. Sherchand, Grant Liao, Mark Mednikov, Agnes L. Chenine, M. Javad Aman, Cory L. Nykiforuk, Rajan P. Adhikari

**Affiliations:** ^1^ Research and Development, Emergent BioSolutions Canada Inc., Winnipeg, MB, Canada; ^2^ Department of Immunology, Max Rady College of Medicine, University of Manitoba, Winnipeg, MB, Canada; ^3^ Integrated Biotherapeutics Inc. (IBT), Rockville, MD, United States

**Keywords:** *S. aureus*, hyperimmune, IBT-V02-F(ab’)2, bacteremia model, pneumonia model, IBT-V02 vaccine

## Abstract

*Staphylococcus aureus* has been acquiring multiple drug resistance and has evolved into superbugs such as Methicillin/Vancomycin-resistant *S. aureus* (MRSA/VRSA) and, consequently, is a major cause of nosocomial and community infections associated with high morbidity and mortality for which no FDA-approved vaccines or biotherapeutics are available. Previous efforts targeting the surface-associated antigens have failed in clinical testing. Here, we generated hyperimmune products from sera in rabbits against six major *S. aureus* toxins targeted by an experimental vaccine (IBT-V02) and demonstrated significant efficacy for an anti-virulence passive immunization strategy. Extensive *in vitro* binding and neutralizing titers were analyzed against six extracellular toxins from individual animal sera. All IBT-V02 immunized animals elicited the maximum immune response upon the first boost dose against all pore-forming vaccine components, while for superantigen (SAgs) components of the vaccine, second and third doses of a boost were needed to reach a plateau in binding and toxin neutralizing titers. Importantly, both anti-staphylococcus hyperimmune products consisting of full-length IgG (IBT-V02-IgG) purified from the pooled sera and de-speciated F(ab’)_2_ (IBT-V02-F(ab’)2) retained the binding and neutralizing titers against IBT-V02 target toxins. F(ab’)_2_ also exhibited cross-neutralization titers against three leukotoxins (HlgAB, HlgCB, and LukED) and four SAgs (SEC1, SED, SEK, and SEQ) which were not part of IBT-V02. F(ab’)_2_ also neutralized toxins in bacterial culture supernatant from major clinical strains of *S. aureus*. *In vivo* efficacy data generated in bacteremia and pneumonia models using USA300 *S. aureus* strain demonstrated dose-dependent protection by F(ab’)_2_. These efficacy data confirmed the staphylococcal toxins as viable targets and support the further development effort of hyperimmune products as a potential adjunctive therapy for emergency uses against life-threatening *S. aureus* infections.

## Introduction

*Staphylococcus aureus* (*S. aureus*) is a Gram-positive bacterium that is responsible for a variety of infections in humans ranging from mild skin infection to invasive diseases like sepsis, endocarditis, and pneumonia, which cause severe morbidity and mortality and pose a major challenge to public health. Annually, *S. aureus* is estimated to cause up to 20,000 deaths and cost approximately $15 billion to the health system in the U.S.A (1, 2). In addition, the widespread antimicrobial resistance among *S. aureus* clinical isolates results in severely restricted treatment options using existing antibiotics (3, 4). Alternative approaches to prevent and treat resistant Staphylococcal infections are urgently needed. Currently, there are no licensed vaccines or biotherapeutics targeting invasive Staphylococcal diseases.

*S. aureus* has a broad arsenal of virulence factors that enhance bacterial *in vivo* fitness by exploiting host nutrients, enabling host colonization, or evading host immune responses (5). *S. aureus* cell surface-associated antigens and toxins are two major groups of targets that have been intensively studied for vaccine and antibody development (6–8). Considerable effort has been undertaken to develop a vaccine or immunotherapeutic targeted at *S. aureus* cell surface-associated antigens including clumping factor A (ClfA) [Pfizer SA4Ag, Veronate], iron-regulated surface determinant protein B (IsdB) [Merck V710], lipoteichoic acid (LTA) [Biosynexus pagibaximab], capsular polysaccharide (CP3 and CP8) [Pfizer SA4-Ag, Nabi StaphVax], and manganese transporter protein C (MntC) [Pfizer SA4-Ag], and yet none has imparted protective immunity in controlled clinical trials (9). Indeed, in one case, the vaccination actually worsened the clinical outcome (10, 11). Collectively, these studies suggest that vaccination of surface antigens might induce pathological immune effects mediated by deleterious CD4 T-cell-dependent interferon-gamma (IFNγ) response (12). Corroborating this evidence, an animal study also demonstrated the role of vaccination against cell-surface antigens in enhancing disease severity in an infective endocarditis model (13). For the second major group of targets, seroepidemiology studies suggested a correlation between higher serum levels of toxin-specific antibodies and favorable clinical outcomes (14, 15). The observation that colonized patients present milder disease outcomes as compared to non-colonized patients demonstrated that pre-existing immunity plays some role in protecting humans against severe staphylococcal infections (16). The studies of anti-staphylococcal antibody repertoire in the general population and convalescent patients had highlighted the importance of antibody responses to leukocidins and superantigens (17, 18). These studies suggest multiple staphylococcal toxins may play a major role in the pathogenesis and impact disease outcome. Therefore, passive immunization with antibodies against major virulence factors, like toxins, should have a beneficial effect for patients having low endogenous levels of antibodies or having difficulties mounting an effective humoral response.

Staphylococcal toxins, in particular the cytolytic and superantigenic toxins, mediate tissue destruction and immune evasion (19, 20), target both innate and adaptive immune systems, and impair a wide range of immune cell functions (21). Alpha-hemolysin (Hla, or alpha-toxin), a beta-barrel pore-forming toxin that damages epithelial and endothelial cells as well as keratinocytes, plays a central role in Staphylococcal pathogenesis (22, 23). Bicomponent leukocidins kill immune cells, including phagocytes, natural killer cells, dendritic cells and T lymphocytes, lyse red blood cells to extract iron, and thus represent major virulent factors for immune disruption (24). Superantigens (SAgs) can cause massive polyclonal activation of T cells leading to cytokine storm and toxic shock, induce intoxication and dysregulation of a variety of specialized T cell subsets, and induce anergy and lymphocyte apoptosis (25). Therapeutic antibodies targeting these toxins could rescue the immune cells, preserve the immune system architecture, and ensure an effective immune response elicited against infection while not interfering with the administration of antibiotics. The anti-virulence approach could also reduce the risk of bacteria’s acquisition of resistance, due to the absence of selective pressure that directly affects bacterial survival.

Several monoclonal antibodies (mAbs) are currently being evaluated in human trials. Tosatoxumab (AR-301, an anti-Hla human IgG1), developed by Aridis, is recruiting for a phase III trial in patients (study #NCT03816956) with *S. aureus* ventilator-associated pneumonia (VAP) in addition to standard of care treatment. Suvratoxumab (AR-320, another anti-Hla human IgG), developed by AstraZeneca and recently licensed by Aridis, is engineered for extended half-life, and has displayed some efficacy in reducing the development of *S. aureus* VAP (phase II trial) (26). X-Biotech has developed 514G3, a mAb against Protein A (SpA), completed a combined phase I/II clinical trial (ClinicalTrials.gov Identifier: NCT02357966) on bacteremia patients, and reported a reduction in *S. aureus*-related adverse effects, but statistical significance was not reached. Overall, mAb immunotherapeutics for *S. aureus* prophylaxis and treatment showed promising clinical outcomes but require further validation in larger controlled clinical studies (27). Given the complexity of *S. aureus* pathogenesis and the diversity of virulent mechanisms, a polyclonal approach can simultaneously neutralize multiple virulent factors and is highly resistant to escape. In fact, intravenous immunoglobulin (IVIG), which contains pooled human polyclonal antibodies, can neutralize Hla and PVL produced by methicillin-resistant *S. aureus* (MRSA) and confer protection in a rabbit model of necrotizing pneumonia (28). However, affinity-purified anti-LukS/F-PV and anti-Hla antibodies from IVIG at 1 mg/kg provided similar protective efficacy as IVIG at 200 mg/kg, which indicated that a hyperimmune approach may be needed for providing higher titer polyclonal antibodies at lower administration volume to achieve the necessary benefits. De-speciated equine polyclonal immune globulin (EIG) is an attractive alternative for IVIG, due to its high antibody yield, ease of management of antibody response, low risk of human virus or adventitious agent contamination, and removal of the Fc region could avoid enhancement effects (mediated through Fc effector function) or aggravate inflammation observed in vaccine clinical trials. The new generation of EIG products containing highly purified F(ab’)_2_ fragments were demonstrated to be safe and well-tolerated in clinical use (29, 30). However, the effect of removal of Fc fragment on Ab-mediated toxin neutralization is inconclusive (31, 32), and the impact of reduced half-life of EIG on toxin clearance needs to be evaluated before proceeding to large-scale EIG manufacture.

In the present study, we immunized rabbits using a multicomponent vaccine IBT-V02, comprised of toxoids for Hla (Hla_H35LH48L_), subunits of PVL (LukS_mut9_, LukF_mut1_), LukAB (LukAB_mut50_), and a fusion of three SAgs (toxoid of TSST-1, SEB, and SEA; TBA_225_) (8). As a surrogate to equine hyperimmune products, we generated rabbit F(ab’)_2_ and performed proof-of-concept studies, which demonstrated that the multivalent antibody approach targeting extracellular toxins provides significant protection against MRSA infection, the anti-Staph F(ab’)_2_ (IBT-V02-F(ab’)2) fragment provided equivalent *in vitro* Ab-mediated toxin neutralization as the full-length IgG (IBT-V02-IgG), and by increasing the bioavailability, IBT-V02-F(ab’)2 is as effective as the full-length IBT-V02-IgG in protecting animals in stringent MRSA infection models.

## Material and Methods

### Bacteria Strains, Toxins, and Cell Lines

*S. aureus* strains: USA300 NRS384, USA100, MN8, Newman, and MNHOCH were received from Network on Antimicrobial Resistance in *S. aureus* (NARSA) (currently BEI, beiresources.org). Previously, these strains had been well characterized for their toxin profile by western blots as well as culture supernatant toxicity in rabbit blood (33, 34). Bacterial strains were further confirmed by the hemolytic pattern on sheep blood agar. Bacterial culture supernatants were prepared in 7 ml BHI media in 50 ml Falcon tubes incubating overnight at 37°C in a shaking incubator (230 rpm). The next day, OD_600_ was measured and normalized to 6.0 using BHI media. Culture supernatants were prepared by centrifuging the cultures followed by sterile filtration passing through 0.2 μm filters. Culture supernatants were aliquoted and stored at -80°C.

For challenge dose preparations, fresh BHI plates were inoculated by a glycerol stock generated from a single colony USA300 NRS384. The next day, colonies were transferred into 7 ml BHI broth and grown overnight to prepare the inoculum. On the 3^rd^ day, 1 liter of BHI broth was inoculated with inoculum and grown for 6 hours at 37°C shaker flask (230 rpm). Culture suspension was then centrifuged, washed in PBS, and frozen stock aliquots were prepared and stored at -80°C.

*S. aureus* toxins (Hla, PVL, LukAB, LukED, HlgAB, and HlgCB) and superantigens (SEA, SEB, SED, TSST-1, SEK, SEQ, and SEC1) were provided by IBT Bioservices (ibtbioservices.com) with > 98% purity. The toxins were reconstituted in PBS and stored at -80°C until use.

The HL-60 cells, from ATCC (atcc.org), Manassas, VA were cultured in RPMI media containing 82 U/ml of penicillin and streptomycin with 16% FBS. Cells were differentiated in media with 1.5% dimethyl sulfoxide (DMSO) as described previously (6, 8, 33). PBMCs from healthy volunteers were obtained from StemExpress (stemexpress.com, Cat #LE010F).

### IBT-V02 Vaccine Components Purification and Characterization

IBT-V02 vaccine consists of five rationally designed toxoid proteins (8) including Hla toxoid, PVL toxoid consisting of mutated S and F subunit proteins, and a dimeric LukAB toxoid. To generate the inactivated toxoids of SEA, SEB, and TSST-1, three-point mutations were introduced in each corresponding wild type toxin (SEA_L48R/D70R/Y92A_, SEB_L45R/Y89A/Y94A,_ and TSST-1_L30R/D27A/I46A_), which are the critical amino acid residues in the MHC binding sites of these toxins. These mutants had previously been tested in different animal models (35). For SEA_L48R/D70R/Y92A_, we introduced another extra mutation H225A to generate SEA_L48R/D70R/Y92A/H225A,_ based on the published literature on the role of this amino acid to activate the T-cell response (36, 37). Individual toxoids were linked together by a flexible linker (three GGGGS repeats (3xG4S)) in order of TSST-1_L30R/D27A/I46A_, SEB_L45R/Y89A/Y94A,_ and SEA_L48R/D70R/Y92A/H225A_. Hence, the name TBA_225_. This construct was expressed in *E. coli*, and the fusion protein was purified by column chromatography as described below as well as in our previously published paper (7).

The individual toxoid proteins were cloned in the vector pET24a+ and expressed in BL21 DE3 *E. coli*. IPTG (0.3 mM) was used to induce the expression of proteins. Cells were lysed by lysis buffer with lysozyme (1 mg/ml) followed by sonication. The supernatant containing the proteins was precipitated with ammonium sulfate. The pellet was then resuspended and desalted into capture column equilibration buffer and loaded over a Poros 50 HS cation exchange column. Fractions were collected and tested to identify the eluted fractions containing the toxoids by running and staining SDS gel against standards. Fractions were then collected and dialyzed. Each protein was thoroughly tested and stored at -80°C in storage buffer. A detailed purification method for different components of IBT-V02 has been published elsewhere (6, 7, 38). The finished products have a high level of purity, very low level of endotoxin, and expected molecular weight as characterized by SEC-HPLC, SDS-PAGE, and other QC methods described in our previous publication (8).

### Rabbit Immunization

**Figure 1:**
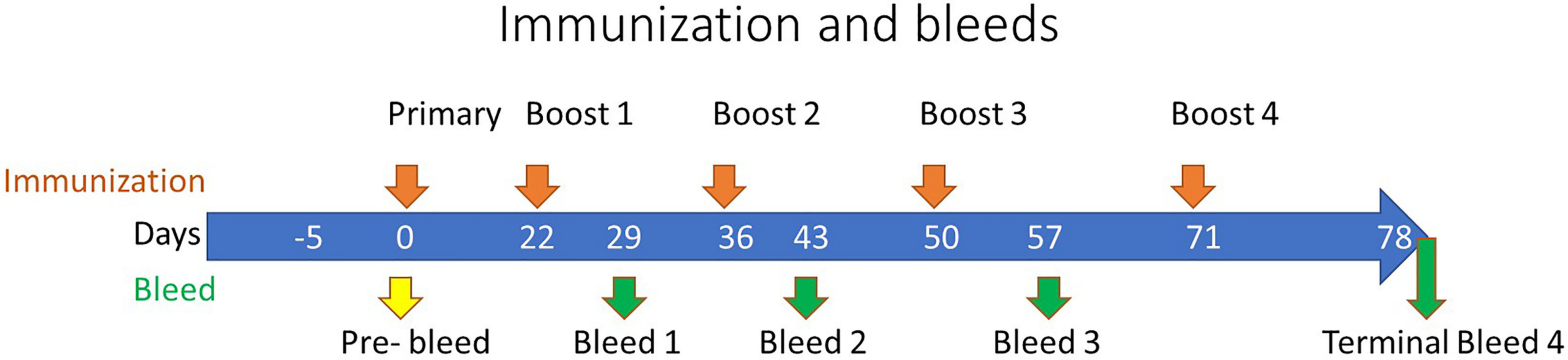
Rabbit Immunization Schedule. The immunization of rabbits is depicted along the top of the timeline by orange arrows at 0, 22, 36, 50, and 71 days representing the primary and subsequent boosts, respectively. The pre-immunization bleed (yellow arrow) was conducted at day zero with subsequent test bleeds performed along the timeline as denoted by the green arrows at 29, 43, and 57 days, with a terminal bleed at day 78.

For rabbit immunizations, five toxoid proteins were blended, based on equal weight ratios, and used as a pentavalent immunogen. Immunogen was emulsified with Complete Freund’s Adjuvant (CFA) for prime and incomplete Freund’s Adjuvant (IFA) for boosts in a 1:1 ratio (v/v) before subcutaneous (s.c.) injection. The immunization dose was 200 µg/animal of total protein (40 µg of each toxoid) for the prime and final push, and 100 µg total protein (20 µg of each toxoid) for boosts. A total of 15 New Zealand White (NZW) specific pathogen-free (SPF) rabbits were immunized 5 times before final bleed to collect serum at Rockland under an IACUC approved protocol. Test bleeds were collected one week after each immunization. The immunization schedule is illustrated in **Figure 1**.

### Antibody Purification and Characterization

After the terminal bleed, the serum from each rabbit was pooled and prepared for Low Endotoxin Unit (EU) purification (performed by Rockland Immunochemicals SOP 073A) using liquid chromatography (Protein A Sepharose 4 Fast Flow; performed by Rockland Immunochemicals SOP 073A). Purified rabbit IgG, meeting specification for purity and low endotoxin, was shipped to Emergent BioSolutions for final preparation of the test articles, IBT-V02-IgG and IBT-V02-F(ab’)2 (EBSI protocol PRO047450).

In general, the purified antibody was dialyzed in PBS buffers and transferred aseptically to the final container. The purified IgG was analyzed by HPLC using a Tosoh TSKgel UP‐SW3000 (4.6 mm x 30 cm) Column on an Agilent 1100, and by SDS‐PAGE under reduced and non‐reduced conditions. Part of purified IgG (9.82 mg/ml) was formulated into 10% (w/w) maltose, 0.03% (w/w) polysorbate 80 (PS80) as final formulation to provide an intact IgG hyperimmune control (IBT-V02-IgG). Another part of purified IgG was digested with pepsin to produce F(ab’)_2_ plus F(ab’)_2_-related immune globulin fragments. The digested material was further purified through diafiltration and anion exchange chromatography. The purified F(ab’)_2_ product was diafiltrated and formulated into 10% maltose and 0.03% PS80 for the F(ab’)_2_ test articles (IBT-V02-F(ab’)2). Sera collected from naïve rabbits were used as a negative control, the same purification process and formulation were used to purify control Rabbit F(ab’)_2_ (RF(ab’)2). For experiments where indicated, purified intact control Rabbit IgG (R-IgG) was purchased from Rockland. Part of the IBT-V02-F(ab’)2 was further polished using EndoTrap^®^ HD resin and columns to meet established specifications for endotoxin levels. Two different lots of final products: lot#1 (Lot#740_POC_17_001_004) and lot #2 (Lot# 510063-20-001*)* were characterized using standard QC methods and the results are summarized in **Table 1**.

**Table 1:** Test Articles and Control Articles with Characterization Parameters. All test articles and controls met specifications for *in vitro* and *in vivo* testing.

Sample	Parameter		Specification	Results	
IBT-V02-F(ab’)2 (Lot#1)**	Protein by A_280_	FIO *	11.10 mg/mL		
	SEC-UPLC	Fab/F(ab’)_2_	≥90%	>97.0%	
		HMW	≤2%	<1.0%	
		LMW	≤7%	<3.0%	
	pH	5.0-6.5	5.49	
	Bacterial Endotoxins	≤5.0 EU/mL	1.766 EU/mg		
	Maltose	9-12 g%	10.066 g%		
	Polysorbate 80	0.10-0.40 mg/mL	0.3069 mg/mL		
	Capillary Gel Electrophoresis	F(ab’)_2_	FIO	97.8%
		Fab +F(ab’)_2_ fragments	FIO	2.2%	
IBT-V02-F(ab’)2 (Lot#2)***	Protein by A_280_	FIO	15.96 mg/mL		
	SEC-UPLC	Fab/F(ab’)_2_		≥90%	94.7%
		HMW		≤2%	0.8%
		LMW		≤7%	4.5%
	Bacterial Endotoxin	≤5.0 EU/mL	0.578 EU/mg		
Control Rabbit Fab/F(ab’)_2_	Protein by A_280_	FIO	11.12 mg/mL		
	SEC-UPLC	Fab/F(ab’)_2_	≥90%	>97.0%	
		HMW	≤2%	<1.0%	
		LMW	≤7%	<3.0%	
	pH	5.0-6.5	5.42		
	Bacterial Endotoxins	≤5.0 EU/mL	0.219 EU/mg		
	Maltose	9-12 g%	10.117 g%		
	Polysorbate 80	0.10-0.40 mg/mL	0.3105 mg/mL		
	Capillary Gel Electrophoresis	F(ab’)_2_	FIO	97.8%	
		Fab +F(ab’)_2_ fragments	FIO	2.2%	
IBT-V02-IgG	Protein by A_280_	FIO	9.82 mg/mL		
	SEC-HPLC (% Monomeric IgG)	FIO	99.1%		
	Bacterial Endotoxin	≤5.0 EU/mL	0.358 EU/mg		

^*^FIO: These results are for information only.

**IBT-V02-F(ab’)2, lot 1 was used for in vivo studies.

***IBT-V02-F(ab’)2, lot 2 was used for in vitro studies.

### Luminex Assay

A multiplex assay to detect serum IgG titers to cytolytic toxins and SAgs has been previously developed at IBT using the Luminex^©^ xMAP technology. Briefly, IBT-V02 target antigens, Hla, LukS-PV, LukF-PV, LukAB, SEA, SEB, and TSST-1, were coupled to carboxylated MagPlex microsphere beads with distinct spectral regions *via* a carbodiimide reaction. Individual bead stocks (bead regions) were mixed in equal ratios to generate a multiplex master mix. The master mix was co-incubated with serum samples in a 96-well plate for 2 hours at room temperature with shaking, washed three times on a magnetic plate washer and co-incubated with a secondary PE-labeled anti-rabbit IgG for detection. Plates were washed, and samples were acquired on a Luminex 200 instrument. Data were analyzed using a 4-parameter logistic (4PL) curve fit in XLFit (IDBS, Microsoft). IgG titers were expressed as the effective dilution at the point of the 4PL curve where 50% (ED_50_) of antigen binding was detected by toxin-specific antibodies present in the serum sample.

### Toxin Neutralization Assay

Hla TNAs were performed as previously described (8). In brief, 8% rabbit red blood cells (RRBCs) were co-incubated with wildtype Hla ± serially diluted serum samples at 37°C. Cells were centrifuged after 30 min and absorbance determined at OD_416nm_. PVL and LukAB TNAs were performed with human promyelocytic leukemia (HL-60) cells as previously described (25). In brief, differentiated HL-60 cells were incubated with either PVL or LukAB ± serially diluted serum samples at 37°C with 5% CO_2_ for 3 hours, and CellTiter Glo was added to the culture to measure cell viability.

For superantigen TNAs, serum or purified antibodies were serially diluted in RPMI 1640 media (Gibco A10491), mixed with EC_98_ concentration of superantigens, and incubated at room temperature for 1 hour in a 96-well plate (BD Falcon BD353376). PBMCs (StemExpress #LE010F) from healthy volunteers were added such that the final density was 1 × 10^5^ cells per well; plates were incubated at 37°C with 5% CO_2_ for 48 hours. The supernatants were collected and IFN-γ was measured by ELISA (R&D Systems #DY285B), to calculate neutralization as described in our previous publications (7, 8). A 4-parameter logistic (4PL) curve fit in XLfit (IDBS, Microsoft) was used to analyze the data. Based on the 4PL plots, ND_50_ (50% neutralizing dilution for serum) or NC_50_ (50% neutralizing concentration for purified antibody) were calculated.

### USA300 MRSA Bacteremia Model

Female BALB/c mice were purchased from Charles River Laboratories. The age of mice for each experiment was 6-8 weeks. Mice were challenged intraperitoneally (IP) with a lethal dose of USA300 NRS384 in 200 µl volume and monitored for health, weight, and survival for 14 days post-challenge. For the full length (IBT-V02-IgG) and F(ab’)_2_ (IBT-V02-F(ab’)2) comparative study, mice were administered IP (150 mg/kg) -2 hours prior to bacterial challenge once for the IBT-V02-IgG group, whereas for F(ab’)_2_ control (RF(ab’)2) and IBT-V02-F(ab’)2, mice were dosed at -2, +24, 48, 72, and 96 hours post challenge in 500 µl volume. Two different challenge doses 1xLD90 and 2xLD90 were used. For IBT-V02-F(ab’)2 dose-response study, we treated mice twice a day in two different doses: (75 mg/kg per dose; 150 mg/kg/day) or (50 mg/kg per dose; 100 mg/kg/day) in two different challenge doses 1xLD90 or 1.5xLD90. Mice were maintained under pathogen-free conditions and fed laboratory chow and water *ad libitum*.

### USA300 MRSA Pneumonia Model

For the pneumonia model, mice (6-8 wks.) were anesthetized with isoflurane and were intranasally (IN) challenged with a lethal dose of *S. aureus* (USA300 NRS384) in 20 or 30 µl DPBS. F(ab’)_2_ was administered two times per day (50 mg/kg, 75 mg/kg, or 100 mg/kg per dose) through IP route. Mice were dosed at days: D0, D1, D2, D3, and D4 post challenge in 250 µl volume per dose. Four different challenge doses were used: 5x10^8^, 4x10^8^, 3.5x10^8^, and 3x10^8^ CFU/mouse. Animals were monitored for survival, health scores, and body weight two times a day (AM and PM) within the first 4 days of challenge and then once a day until termination of the study (day 14) as described previously (39).

### Statistical Analysis

Data were analyzed using GraphPad PRISM v8.4.3. Statistical analysis was performed using an unpaired t-test for the comparison of area under the curve (AUC) for supernatant-based antibody neutralization assays with the controls. An overall significance level of 5% was used and the probability of a type 1 error for each test was adjusted for multiple comparisons. Kaplan-Meier curves along with log-rank (Mantel-Cox tests) were used to compare the survival rates for each treatment group to the negative control group.

## Results

### IBT-V02 Vaccine Component Is Highly Immunogenic and High Titer Antibodies Were Generated Against Vaccine Components

**Figure 2:**
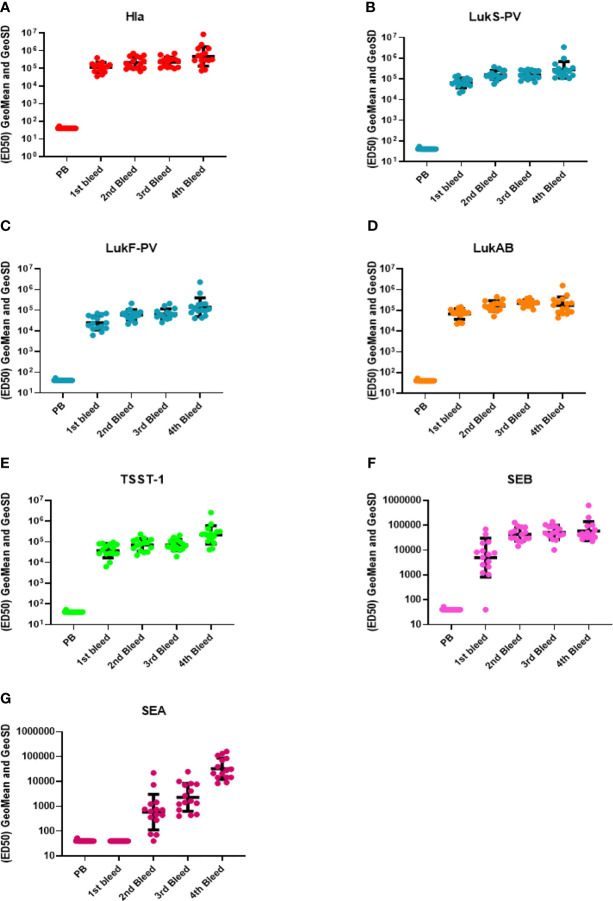
Antibody titers during rabbit immunization. The 50% effective dilution (ED_50_) for the antigen binding determined by the Luminex assay whereby 50% of antigen was detected by toxin-specific antibodies present in the serum sample, was measured in the pre-bleed (PB) and three test bleeds along with the fourth and final terminal bleed (refer to **Figure 1**). Toxin components are **(A)** Hla, **(B)** LukS-PV, **(C)** LukF-PV, **(D)** LukAB, **(E)** TSST-1, **(F)** SEB, and **(G)** SEA. Geomean values (black lines) were plotted along with GeoSD.

IBT-V02 vaccine consists of five rationally designed proteins, seven fully attenuated toxoids, and can elicit immune responses against over 15 Staphylococcal toxins by homological cross-neutralization (8). Hla toxoid harbors two mutations (H35L and H48L), PVL toxoid consists of mutated S and F subunit proteins (LukS_mut9_ and LukF_mut1_), a dimeric toxoid LukAB_mut50_, and a fusion protein TBA_225_, which represents three superantigen toxoids fused together *via* a flexible linker (TSST-1, SEB and SEA). Each of these toxoids is fully attenuated, and the safety and immunogenicity had been extensively studied previously (40). Using five toxoid proteins blended at equal weight ratios as immunogen to immunize rabbits, strong antibody responses were elicited. Sera from test bleeds were collected one week after each boost (**Figure 1**) and antibody titers to seven IBT-V02 targeted antigens (Hla, LukS-PV, LukF-PV, LukAB, SEA, SEB, and TSST-1) as determined by a multiplex Luminex assay for each rabbit are presented as the effective dilution of the sera that produced 50% maximal response (ED_50_) (**Figure 2**). IBT-V02 induced robust antibody responses after the first boost to all the antigens except SEA. The average ED_50_ titer for Hla is 136,673, LukS-PV is 72,458, LukF-PV is 32,083, LukAB is 77,572, SEB is 14,369, and TSST-1 is 47,006. The magnitude of the responses to each antigen was comparable to the titers obtained from previous studies, except for SEA (8). However, the second boost did induce antibody response to SEA, and the titers steadily increased until the last terminal bleed, reaching an average ED_50_ titer of 50,385. The antibody titers to other antigens were also gradually increased upon subsequent boosts. The terminal bleed titers against all target antigens were significantly higher than the assay internal control, which consisted of antibodies obtained by immunizing rabbits with IBT-V02 two times (IBT generated rabbit polyclonal sera). Individual rabbit responses were variable, but in close range against each antigen. There were no “non-responders” observed in the group, indicating that IBT-V02 is highly immunogenic and can successfully induce universal responsiveness with a multivalent approach.

### Anti-Staph Rabbit Antibodies Are Potent Against Staphylococcal Toxins

**Figure 3:**
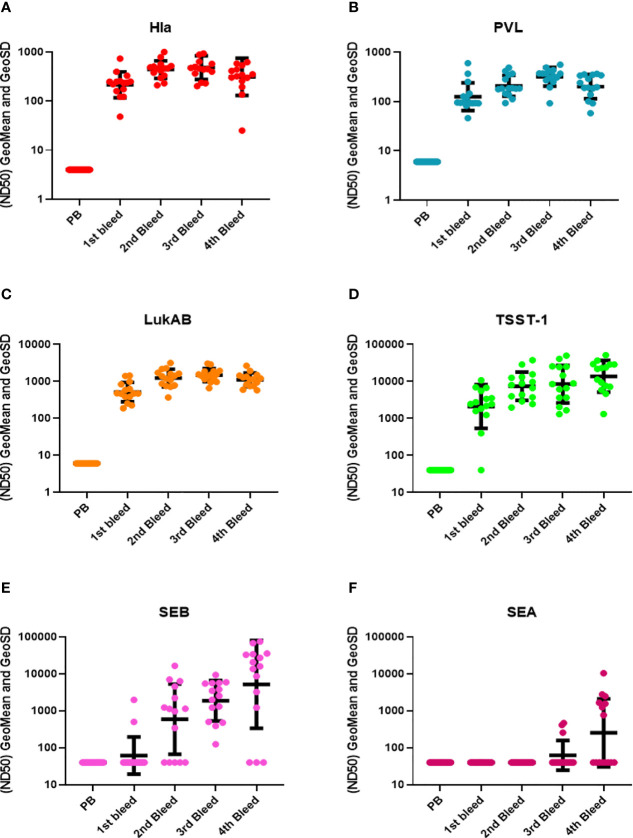
TNA titers during rabbit immunization. The 50% neutralizing dilution (ND_50_) for six different toxins were measured as described in materials and method sections. Neutralizing antibody titers (ND_50_s) were measured in the pre-bleed (PB) and three test bleeds along with the 4^th^ and final terminal bleed (refer to **Figure 1**). Toxins included were **(A)** Hla, **(B)** PVL (LukS+LukF), **(C)** LukAB, **(D)** TSST-1, **(E)** SEB, and **(F)** SEA. Geomean values (black lines) were plotted along with GeoSD.

Three types of toxin neutralization assays (TNA) were used to further examine the function of antibodies elicited by IBT-V02 immunization. The potency of antibodies against Hla was measured by the inhibition of alpha-toxin dependent rabbit red blood cell lysis. The potency against PVL and LukAB was determined by the inhibition of leukocidin-induced cytotoxicity in PMN-like HL-60 cells. Human PBMC based superantigen TNAs were carried out to study the antibody potency against SEA, SEB, and TSST-1. As observed in the Luminex titers (**Figure 2**), the first boost elicited strong neutralizing antibodies to Hla, PVL, LukAB, SEA, SEB, and TSST-1, with few exceptions where neutralizing antibodies were not generated against SEA. The second boost increased the antibody response to all the antigens except SEA (**Figure 3**). The response to Hla, PVL, LukAB, and TSST-1 plateaued after the second boost. The response to SEB increased by subsequent boost, and upon the last boost, the neutralization titer was significantly increased for SEA. Taken together, the binding and neutralization studies showed that highly potent antibodies against all vaccine components were generated by IBT-V02 immunization.

### IBT-V02- F(ab’)2 and IBT-V02-IgG Have Equivalent Potency in *In Vitro* Toxin Neutralization

**Figure 4:**
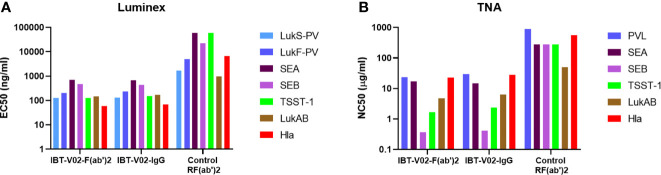
Anti-Staph IBT-V02-F(ab’)2 and IBT-V02-IgG binding and neutralization potency were compared with the control RF(ab’)2. **(A)** Luminex binding titers were compared based on 50% effective concentration (EC_50_) for seven antigens (components of IBT-V02), Hla, LukS-PV, LukF-PV, LukAB, SEA, SEB, and TSST-1. **(B)** Toxin neutralizing titers were compared based on 50% neutralizing concentration (NC_50_) for six toxins, Hla, PVL (LukS-PV + LukF-PV), LukAB, SEA, SEB, and TSST-1. EC_50_ and NC_50_ values were determined using a 4PL curve fit in XLFit (IDBS, Microsoft). Since no significant difference was noted among the tested two lots, data from one lot were plotted as a representation.

Next, we sought to characterize the anti-Staph hyperimmune products and compare the profiles of full-length IgG and de-speciated F(ab’)2 derived from the pooled sera of all 15 immunized rabbits. In this series of experiments, IBT-V02-IgG and IBT-V02-F(ab’)2 antibody products were tested for their binding and neutralization potencies. The Luminex EC_50_ for antibodies against all antigens were in hundreds of ng/mL (**Supplementary Table S1**) range, significantly lower than the control rabbit F(ab’)_2_ (RF(ab’)2) generated from naïve animals. Purified antibody products also exhibited strong neutralization against toxins. The TNA NC_50_s were in the single or double µg/mL range (**Supplementary Table S2**). When the binding and neutralization potencies were compared among the two IBT-V02-F(ab’)2 lots and one full-length IgG (IBT-V02-IgG) lot, there was no significant difference among different antibody products, indicating that the removal of Fc region did not affect IBT-V02-F(ab’)2 *in vitro* potency (**Figure 4**).

### IBT-V02- F(ab’)2 Cross-Neutralizes Other Leukocidins and Superantigens

**Figure 5:**
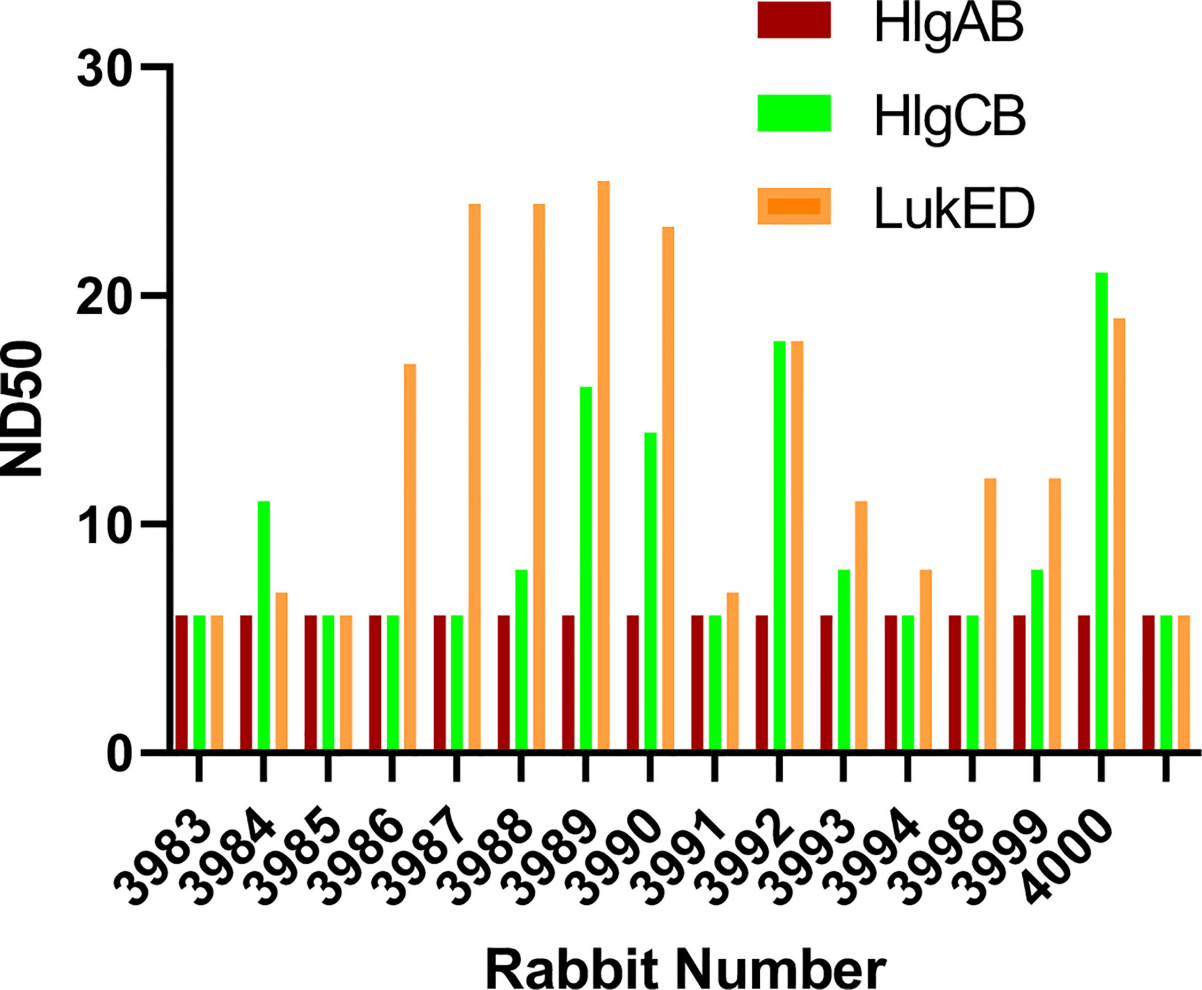
The cross-neutralization of terminal bleeds against other leukocidins. All 15 rabbit samples from terminal bleed were run in full curve and 50% neutralizing dilutions (ND_50_) were determined for three leukotoxins: HlgAB, HlgCB, and LukED.

**Figure 6:**
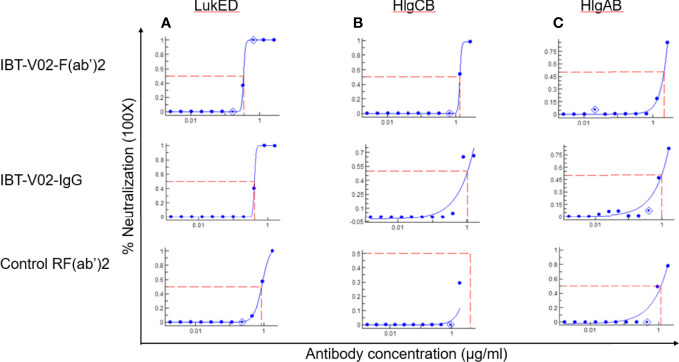
Cross neutralization data against 90% effective concentration (EC_90_) of **(A)** LukED, **(B)** HlgCB, and **(C)** HlgAB by IBT-V02-F(ab’)2, IBT-V02-IgG and negative control RF(ab’)2 tested in full curve and analyzed by a curve fit in XLFit (IDBS, Microsoft).

**Figure 7:**
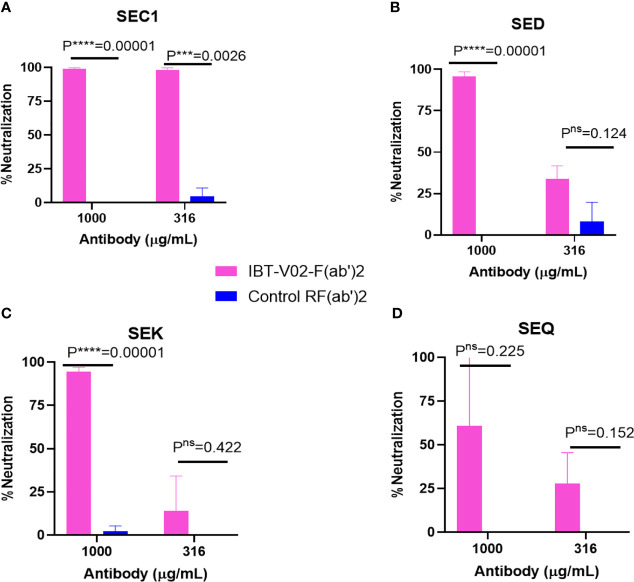
Cross neutralization data against 90% effective concentration (EC_90_) of **(A)** SEC1, **(B)** SED, **(C)** SEK, and **(D)** SEQ by IBT-V02-F(ab’)2 and control RF(ab’)2 tested in two different concentrations (1,000 and 316 µg/ml) of antibodies. The data were analyzed by unpaired multiple t-tests.p- values are represented as: ns, P > 0.05; ***, P ≤ 0.001; ****, P ≤ 0.0001.

The bicomponent leukocidins produced by *S. aureus* include PVL, gamma hemolysins (HlgAB and HlgCB), LukED, and LukAB. The S and F subunits of bicomponent leukocidin have a high degree of homology respectively across the family except for LukAB. *S. aureus* also produces more than 20 types of SAgs, which have limited primary sequence identity but exhibit considerable structural homology. IBT-V02 vaccine contains toxoids for SEA, SEB, and TSST-1, which represent three divergent subgroups of SAgs. We tested the cross-neutralization potential first in terminal bleed samples from individual animals against leukotoxins (**Figure 5**) and then in purified antibodies (**Figure 6**). To further evaluate the toxin neutralization ability of newly purified anti-Staph hyperimmune product, we examined the cross-neutralization of IBT-V02-F(ab’)2 against leukocidins and superantigens that were not part of the vaccine components. Results showed that IBT-V02-F(ab’)2 exhibited neutralization against HlgCB and LukED when compared to control RF(ab’)_2_, (**Figure 6**) with the NC_50_ values in the μg/ml range, so the potency was around 10 times lower than that against PVL (**Supplementary Tables S2**,**S3**). The superantigens SEC1, SED, SEK, and SEQ were used to investigate the cross-neutralization ability of IBT-V02-F(ab’)2 against superantigens, and results indicated that IBT-V02-F(ab’)2 can totally neutralize SEC1 at two concentrations tested, and neutralize SED and SEK in higher concentrations, but to a lesser extent against SEQ (**Figure 7**). These data demonstrated that the cross-neutralizing activity of anti-Staph hyperimmune product can be achieved by a rationally designed multivalent vaccine with specific strategies targeting homology and specificity.

### IBT-V02-F(ab’)2 Recognizes Toxins Produced by *S. aureus* Clinical Isolates

**Figure 8:**
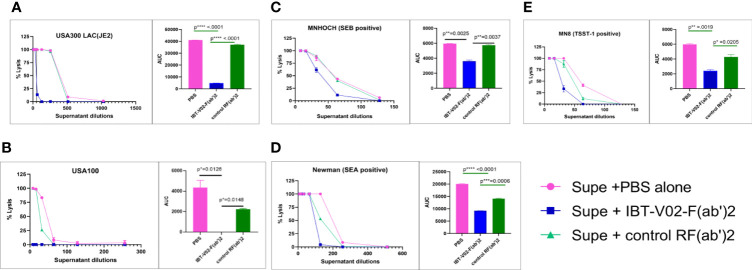
The bacterial culture supernatant neutralization by IBT-V02-F(ab’2) in RRBC hemolytic assay. **(A)** USA300 LAC JE2, **(B)** USA100, **(C)** MNHOCH, **(D)** Newman, and **(E)** MN8 neutralization by F(ab’)2. In each figure, the AUC is shown as an inset. The data were analyzed by unpaired t-test. p- values are represented as: ns, P > 0.05; *, P ≤ 0.05; **, P ≤ 0.01; ***, P ≤ 0.001; ****, P ≤ 0.0001

*S. aureus* strains in different clonal complexes display considerable variability in toxin profiles. The neutralizing potentials of IBT-V02-F(ab’)2 were compared in bacterial culture supernatant from different clinical strains with control RF(ab’)2 and PBS alone. Among the selected strains were the USA300 LAC (JE2), USA100, and *S. aureus* strains which are positive for SEB, SEA, and TSST-1 superantigens. As shown in **Figure 8**, supernatant toxicity (PBS group) varies widely among the strains, and as expected, USA300 LAC exhibited the highest toxicity (rabbit blood-based hemolysis assay) followed by SEA positive Newman strain. We observed ED_50_s of 400; 150; 64; 50; and 40 for JE2 (USA300, LAC), Newman, MNHOCH, MN8, and USA100, respectively. IBT-V02-F(ab’)2 neutralizing activities were most prominent in USA300 LAC and USA100 strains when compared with MN8, Newman, and MNHOCH in presence of 40 µg/ml when compared with Control-RF(ab’)2 (**Figure 8**). AUC showed a significant reduction in toxicity in (IBT-V02-F(ab’)2) when compared either with PBS alone or with control RF(ab’)_2_ (**Figure 8** AUC bar diagrams inset figures next to each plot). For supernatant neutralization of superantigens, PBMC based assays demonstrated that 100 µg/ml of IBT-V02-F(ab’)2 significantly decreased the amount of IFNγ production (readout of superantigenicity) when compared with either media alone or with control RF(ab’)2 (**Supplementary Figure S1**) in all three tested strains: MN8 (TSST-1); Newman (SEA); and MNHOCH (SEB). Similarly, as proof of concept, we selected two bacterial culture supernatants, JE2 and Newman, to measure the neutralizing potency of IBT-V02-F(ab’)2 against leucotoxins. As shown in **Supplementary Figure S2**, IBT-V02-F(ab’)2 significantly decreased the % lysis in HL-60 based assays. Nevertheless, cross-neutralizing activities of specific IBT-V02-F(ab’)2 were clearly demonstrated in all clinical isolates tested.

### IBT-V02-F(ab’)_2_ Protects Against USA300 MRSA Infection in Bacteremia Model

**Figure 9:**
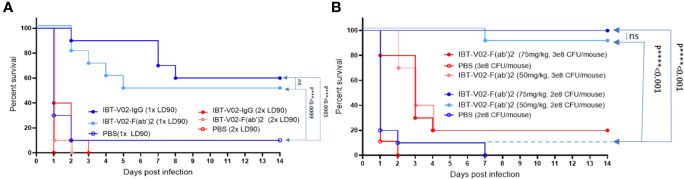
Efficacy studies in BALB/c mice challenged with USA300 (NRS384) in bacteremia model. **(A)** Comparison of IBT-V02-IgG and IBT-V02-F(ab’)2 either at 1xLD90 or at 2xLD90 with different antibody treatment doses. **(B)** Different treatment doses of IBT-V02-F(ab’)2 are compared with two different challenge doses. The data were analyzed by log-rank (Mantel-Cox) test. ns, not significant; ***, P < 0.001; ****, P < 0.0001.

Given the equivalent *in vitro* potency observed for IBT-V02-F(ab’)2 and IBT-V02-IgG, we investigated whether these antibody products protected in a murine model of MRSA-induced bacteremia and could demonstrate equivalent *in vivo* potency. The bacteremia was established by intraperitoneal injection of USA300 strain NRS384. Upon 1 x 10^8^ CFU/mouse inoculation, high bacterial loads could be recovered from the liver, kidneys, spleen, and lungs 2 days after infection, with bacteria disseminated to blood by day 4 and could still be recovered from internal organs 6 days after infection (data not included). The LD90 was determined as 2 x 10^8^ CFU/mouse, which caused 90% mortality among the five inoculation doses tested. A single dose of 150 mg/kg of IBT-V02-IgG at 2 hours before infection and five consecutive doses of IBT-V02-F(ab’)2 at 2 hours before, and 1 day, 2 days, 3 days, and 4 days post-infection (dpi) were given to animals infected with 1xLD90 or 2xLD90 USA300 NRS384 along with the control. Upon 2xLD90 infection, all control group animals died within 24 hours, and only 10% of survival was observed for each treated group. When animals were challenged with 1xLD90 of USA300 NRS384, 90% of control group animals were dead within 48 hours; IBT-V02-IgG provided 60% survival, while IBT-V02-F(ab’)2 protected 50% of animals at the end of the 14-day study. Compared to the gradually decreased survival in the RF(ab’)2 treated group, IBT-V02-IgG treated group maintained 90% for 7 days (**Figure 9A**), which is about the half-life of the rabbit antibody in the murine model (41). When we closely examined the health scores, we found that the animals in the IBT-V02-F(ab’)2 group had lower health scores (i.e., healthier) in the afternoon after treatment compared to their conditions in the morning before treatment, when most mortality occurred. Based on previous studies, we have found that the half-life of other F(ab’)_2_ hyperimmunes in mice is about 5 hours (unpublished data). In comparison, full-length or intact IgG hyperimmunes typically have a half-life of ~3-4 days in mice (unpublished data). Taken together, we hypothesized that the protection could be improved by increasing the bioavailability of IBT-V02-F(ab’)2. To test this hypothesis, animals were treated with IBT-V02-F(ab’)2 or RF(ab’)2 control twice per day, 8 hours apart for 5 consecutive days after 1xLD90 or 1.5xLD90 infection, at two treatment doses, 75 mg/kg and 50 mg/kg. Interestingly, even the lower dose at shorter treatment intervals significantly protected the animals, with 100% and 90% of survival observed against the 1xLD90 challenge (**Figure 9B**). The treatment only provided 20% survival upon 1.5xLD90 infection, but significantly increased the mean time to death (MTD) of treated groups compared to control group (Log-Rank, Mantel-Cox test). The dose and bioavailability dependent protection provided by IBT-V02-F(ab’)2 treatment strongly confirmed the *in vivo* potency of de-speciated anti-toxin antibodies against MRSA bacteremia. The survival data were also reflected in the health score and change in weight data (**Supplemental Figures: S3A, B**).

### IBT-V02-F(ab’)2 Protects Against USA300 MRSA Infection in Pneumonia Model

**Figure 10:**
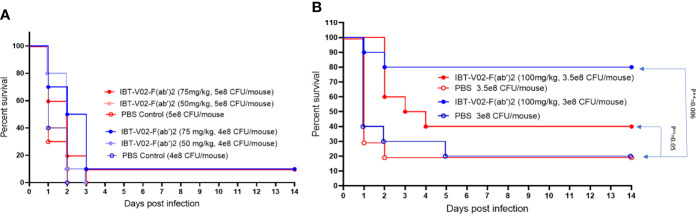
The dose-dependent efficacy studies in BALB/c mice challenged with USA300 (NRS384) in pneumonia model. **(A)** High challenged dose (5 x 10^8^ CFU/mouse and 4 x 10^8^ CFU/mouse) **(B)** Titrated challenge dose (3.5 x 10^8^ CFU/mouse and 3 x 10^8^ CFU/mouse). The data were analyzed by log-rank (Mantel-Cox) test. ns, P > 0.05; **, P ≤ 0.01.

To further evaluate the *in vivo* potency of anti-toxin hyperimmune products, we investigated the protective efficacy of IBT-V02-F(ab’)2 in a mouse pneumonia model. LD90 for this model was not accurately determined due to the observation of either 100% mortality upon higher inoculation or 100% survival upon lower inoculation. Therefore, we tested the efficacy using four different interpolated doses of USA300 strain NRS384 *via* intranasal challenge (**Figures 10A, B**). Upon 5x10^8^ CFU/mouse challenge, only 10% of 75 mg/kg IBT-V02-F(ab’)2 treated animals survived, no significant protection was observed. Upon 4x10^8^ CFU/mouse challenge, 75 mg/kg of treatment significantly extended the MTD but didn’t significantly improve the survival rate (**Figure 10A**). When animals were infected with 3x10^8^ CFU/mouse USA300 NRS384, 100 mg/kg of IBT-V02-F(ab’)2 treatment provided an 80% survival rate, significantly higher than the control group. With the challenge dose increased to 3.5x10^8^ CFU/mouse, the 40% survival was not significant compared to control, but the treatment significantly extended the MTD (**Figure 10B**). The survival data also correlate with the health score and change in weight data (**Supplemental Figures: S4A, B**). These results further confirmed the function of IBT-V02-F(ab’)2 in stringent pneumonia models and validated the efficacy of an anti-toxin passive immunization strategy against *S. aureus* MRSA infection.

## Discussion

In this study, we have presented for the first time that an F(ab’)_2_ hyperimmune generated against multivalent Staphylococcal toxoid vaccine provided significant protection against USA300 MRSA infection in both bacteremia and pneumonia mouse models. The de-speciated rabbit product (IBT-V02-F(ab’)2) retained the *in vitro* binding and neutralizing efficacy against IBT-V02 target toxins compared with full-length IgG (IBT-V02-IgG) and provided similar or even better protection against *in vivo* infection when the product’s bioavailability was improved.

De-speciated equine hyperimmune products instead of full-length IgG have been developed as therapeutics against bacterial toxins (Botulism antitoxin heptavalent types A-G, Emergent BioSolutions). In addition, antibody fragments instead of full-length IgG have been developed to enhance pharmacokinetic properties and/or the efficiency of penetration into tissues or tumor masses (caplacizumab, ranibizumab, abciximab, certolizumab pegol) (42). When the hyperimmune products were generated from heterologous hosts, the IgG Fc fragment was also removed to reduce the incidence of hypersensitivity reactions (43). Currently, there are six anti-toxin F(ab’)_2_ hyperimmune products that are licensed by the FDA, including Botulism antitoxin heptavalent generated from horses, and five antivenin hyperimmunes generated from horse or sheep (44). However, even with the clinical efficacy in these approved products, antibody Fc-mediated clearance is thought to play an important role in toxin neutralization, owing to both effective immune complex (IC) clearance mediated by Fcγ-receptor (FcγR) (45, 46) and IgG stabilization in the bloodstream contributed by the neonatal FcR (FcRn). In BoNT toxicity studies, the antitoxin agents that do not bear Fc had a much shorter half-life and exhibited reduced efficacy due to rapid clearance from the bloodstream (32, 47). Nevertheless, Mazuet et al. (31) showed one mAb (F(ab’)_2_ displayed a much higher affinity and retained its neutralization efficacy compared with intact IgG mAb (31). This evidence suggested that the high affinity of F(ab’)_2_ may compensate for the lack of Fc-mediated mechanisms and may induce neutralization solely *via* toxin blocking. In our study, the anti-staphylococcal toxins F(ab’)_2_ generated by immunization with IBT-V02, have a high binding affinity (**Figure 4A** and **Supplementary Table S1**) and high anti-toxin potency (**Figure 4B**), as indicated by *in vitro* characterization results (**Figures 4A, B** and **Supplementary Tables S1, S2**). In addition, when the daily dose of F(ab’)2 was split into two administrations to maintain the F(ab’)2 steady-state concentration, it improved the bioavailability, hence full protection was observed by IBT-V02-F(ab’)2. Whereas only 60% of protection was observed in the full-length IgG in the MRSA bacteremia model (**Figures 9A, B**). Our data successfully demonstrated that the efficacy of F(ab’)_2_ against toxins could be achieved by improving potency and bioavailability.

The pathogenesis of *S. aureus* is mediated by a vast array of surface-associated proteins, carbohydrate structures, and secreted virulence factors. The complexity of diseases and multiplicity of components pose a significant challenge for the selection of viable targets for active and passive immunization. Previous passive immunization approaches had evaluated a number of surface-associated antigens as targets, including type 5 and type 8 capsular polysaccharide (CPS) (Altastaph), fibrinogen-binding proteins clumping factor A (ClfA) (Veronate and tefibazumab), lipoteichoic acid (LTA) (Pagibaximab), ATP-binding cassette (ABC) transporter GrfA (Aurograb), poly-N-acetylated glucosamine (PNAG) (SAR279356). Unfortunately, these approaches have all failed in clinical trials. Additionally, no clinical success was obtained to date when cell surface-associated targets were developed as vaccine candidates. In fact, in the Phase III clinical trial of V710, a vaccine that targets iron-regulated surface determinant B (IsdB), vaccine recipients were five times more likely to die than the unvaccinated patients when they developed postoperative S. *aureus* infection (11). When cell surface-associated antigens were targeted by antibody-based strategies, phagocytic uptake and/or opsonophagocytic killing were used as indicators to demonstrate the antibody efficacy, despite the fact that no clinical studies have established such outcome as correlate of immunity (48). In the current study, we have taken an alternative approach, which is to target important extracellular secreted toxins to mitigate host tissue damage and prevent toxin-induced immune dysregulation, combined with the removal of the Fc region to avoid enhancement effects observed in clinical trials aimed at inducing direct bacterial killing.

In our study, two major types of virulence factors, pore-forming toxins (PFTs) and superantigens (SAgs), were selected as immunogens to produce the hyperimmune products with the desired function. The multivalent toxoid vaccine IBT-V02 targets Hla, PVL, LukAB, and three superantigens (SEA, SEB, and TSST-1). Hla has been recognized as a key virulence factor of *S. aureus* skin and soft tissue infections (SSTI), bacteremia/sepsis and pneumonia (49, 50) and is currently being evaluated as a therapeutic target in clinical development (27). PVL and LukAB are two of the five leukocidins produced by *S. aureus* isolates that infected humans, the other three are γ-hemolysins AB (HlgAB) and CB (HlgCB) and leukocidin ED (LukED). Leukocidins target an array of human leukocytes that are critical for innate immune defenses (21) and adaptive immunity and are associated with invasive infections such as necrotizing pneumonia (51, 52) and sepsis. Leukocidins have two subunits that are classified as the host cell targeting S component and the polymerization F component. Different from LukAB, which is pre-assembled as a heterodimer, the other S and F components are secreted as monomers before the receptor binding, assembly, and polymerization, and display 68 to 80% amino acid sequence homology. LukA and LukB share only 30 and 40% homology with the other S- and F-components respectively (53–55). Hence, we used toxoid proteins of LukF-PV and LukS-PV components separately and in combination with LukAB as immunogens, with the expectation of generating antibodies with cross-neutralization activities to other PFTs. In fact, we found that the IBT-V02-F(ab’)2 has neutralization activities against HlgAB, HlgCB and LukED (**Supplementary Table 3**), indicating that the anti-Staph hyperimmune generated from the IBT-V02 vaccine comprised of antibodies recognizing all five bicomponent PFTs.

There are more than 29 types of *S. aureus* superantigens (SAgs) in various strains, including staphylococcal enterotoxins (SEs), staphylococcal enterotoxin-like toxins (SEls), and toxic shock syndrome toxin-1 (TSST-1). Most virulent *S. aureus* isolates produce at least one or more SAgs (56). SAgs are not only important for toxic shock syndrome but also play a role in sepsis and possibly other *S. aureus* invasive diseases (57, 58). According to the homology of nucleotide and amino acid sequences, SE and SEI toxins can be classified into several groups, such as the SEA group, SEB group, and SEI group (59). Based on the genetic diversity and the clinical prevalence, the IBT-V02 vaccine includes toxoids of three SAgs (TSST-1, SEB, and SEA) expressed as a fusion protein. When IBT-V02 was used to immunize the rabbits, we found that antibodies raised against SEA and SEB were delayed in comparison to the other vaccine components. That is interesting since high titer antibodies against SEA and SEB were generated upon the first immunization in NHP (40). As immunogenicity is dependent upon the nature of the antigen and species-specific processing and presentation with the host’s MHC molecule on the surface of Antigen Presenting Cells (APCs), we suspect that SAgs are more immunogenic to NHPs, since they are closely related to humans, who are very susceptible to SAgs (60, 61). Nonetheless, after hyperimmunization, high titer antibodies were generated against SEB and SEA, and the potency (NC_50_) of the final product IBT-V02-F(ab’)2 is in the single or double nanomolar range, comparable with some of the anti-SAg mAbs (62, 63). Despite differences in amino acid sequence homology among SAg subgroups, these toxins share high similarity of secondary and tertiary structures (7). The combination of the three most prevalent SAgs also results in an extended cross-neutralization activity (as shown by **Figure 7**) and may provide broad protection against the effects of major SAgs, such as SEC1, SED, SEK and SEQ. These cross-neutralization activities may not be extended to each completed group because of the high molecular diversity of SAgs, but are more relevant for the USA300 epidemic strains where SEQ and SEK SAgs are more prevalent (64).

The multivalent approach we used in this study is not only for neutralizing multiple toxins, but also to provide broad strain coverage. *S. aureus* has a myriad of virulence factors and many of the genes are located on mobile genetic elements (MGE), which can be easily transferred horizontally between strains, causing diverse toxin profiles of different strains (65). On the other hand, many of the virulence factors have some degree of functional redundancy, and the expression is regulated by a complex regulation network that allows bacterial adaptation and survival in the host (66, 67). Various studies have linked specific *S. aureus* clonal groups with particular infections or specific virulence factors (68), but the toxin profiles of *S. aureus* strains are quite heterogeneous, varying remarkably even within each clonal group (69, 70). Given the genotypic and phenotypic diversity of the *S. aureus* strains, we tested the neutralization ability of IBT-V02-F(ab’)2 directly against the toxins present in the bacterial culture supernatants and found that IBT-V02-F(ab’)2 can neutralize the toxins produced by diverse clinical isolates. Furthermore, significant efficacy against USA300 strain was observed (**Figure 8A** and **Supplementary Figure S2**) in rabbit RBC based as well as HL-60 PMN based toxin neutralization assays. The tested USA300 clinical isolate belongs to clonal complex 8 (CC8) group and is the current epidemic lineage and predominant type of MRSA in the United States (71). The strains from this group often produce high levels of cytotoxins and are responsible for severe infections and invasive diseases. Therefore, we used a strain of USA300 MRSA in the animal studies, and results clearly indicate that IBT-V02-F(ab’)2 can provide significant protection against virulent MRSA infection in stringent infection models (**Figures 9**, **10**).

Vaccines could play a pivotal role in the prevention of disease in populations at high-risk for *S. aureus* infection. IBT-V02 has been developed for preventing recurrent SSTI and has shown excellent preclinical efficacy (8). In the current study, we studied whether passive immunization with antibody products could also be applied in acute care treatment settings when there is not enough time for developing an effective immunological response, such as in bacteremia and pneumonia infections, or for vulnerable segments of the population recalcitrant to vaccine response, immunosuppressed or immunocompromised. In the treatment of *S. aureus* infections, we envision that the IBT-V02-F(ab’)2 protects cell and tissue integrity by neutralizing Hla, rescues immune cell function by disabling PFTs, and ensures the balanced response by obstructing SAg-induced over inflammation. IBT-V02-F(ab’)2 could be developed as adjunctive therapy to the standard of care (SOC) antibiotics to improve the clinical efficacy. Therefore, in future studies, we would test combining IBT-V02-F(ab’)2 with SOC antibiotics and examine the synergy and efficacy. Furthermore, the ability to test the combination of treatment and co-vaccination could be explored to prevent recurrence.

In summary, we generated anti-staphylococcus hyperimmune using multivalent IBT-V02 as an immunogen, demonstrating that the de-speciated F(ab’)_2_ (IBT-V02-F(ab’)2) retained the binding and neutralizing efficacy compared to full-length IgG, and also exhibited cross-neutralization titers against other homological toxins. IBT-V02-F(ab’)2 had broad strain coverage against toxins produced by different clinical isolates. The *in vivo* efficacy data generated in bacteremia and pneumonia models using USA300 *S. aureus* strain demonstrated a dose-dependent protection. These efficacy data confirmed that staphylococcal toxins are viable targets and support the further development effort of hyperimmune products as a potential adjunctive therapy for emergency uses against life-threatening *S. aureus* infections.

## Data Availability Statement

The original contributions presented in the study are included in the article/**Supplementary Material**. Further inquiries can be directed to the corresponding authors.

## Ethics Statement

The animal study was reviewed and approved by IACUC.

## Author Contributions

All authors were directly involved in designing the experiments, data analysis, and drafting the manuscript. RO, IM, ThK, TuK, SS, GL, MM, and AC performed experiments. XH, MA, CN, and RA were involved in supervision, data analysis, and writing manuscript. All authors contributed to the article and approved the submitted version.

## Funding

Part of this research was funded by grant from National Institute of Allergy and Infectious Diseases (NIAID) (R43AI136143) to RA.

## Conflict of Interest

RA and MA have stocks in Integrated Biotherapeutics Inc. RO, ThK, TuK, SS, GL, AC, MA, and RA are the currently employed by IBT. IM and MM were IBT employees when the study was conducted. Similarly, CN is currently employed by Emergent, and XH was employed by Emergent when the data was generated. Both have stock options in Emergent BioSolutions.

## Publisher’s Note

All claims expressed in this article are solely those of the authors and do not necessarily represent those of their affiliated organizations, or those of the publisher, the editors and the reviewers. Any product that may be evaluated in this article, or claim that may be made by its manufacturer, is not guaranteed or endorsed by the publisher.
