# Supplementary material for: Hyperimmune Targeting Staphylococcal Toxins Effectively Protect Against USA 300 MRSA Infection in Mouse Bacteremia and Pneumonia Models
Source: Front Immunol. 2022 May 17;13:893921. doi: 10.3389/fimmu.2022.893921 (PMC9152286; doi:10.3389/fimmu.2022.893921)
Supplement: Supplementary file 1 [file DataSheet_1.pdf]

frontiers

Hyperimmune Targeting Staphylococcal Toxins Effectively Protect against USA 300 MRSA Infection in Mouse Bacteremia and Pneumonia Models

Xiaobing Han[1,3], Roger Ortines[2], Ipsita Mukherjee[2], Tulasikumari Kanipakala[2], Thomas Kort[2], Shardulendra P. Sherchand[2], Grant Liao[2], Mark Mednikov[2], Agnes Chenine[2], M. Javad Aman[2], Cory L. Nykiforuk[1*], and Rajan P. Adhikari[2*]

[1]Emergent BioSolutions Canada Inc., Winnipeg, MB, R3T 5Y3, Canada

[2]Integrated Biotherapeutics Inc. (IBT), Rockville, MD 20850, USA

[3]Department of Immunology, Max Rady College of Medicine, University of Manitoba, Winnipeg, MB, Canada

*** Correspondence:**
Cory L. Nykiforuk and Rajan P. Adhikari
cnykiforuk@ebsi.com; rajan@IntegratedBiotherapeutics.com

Supplementary Figures

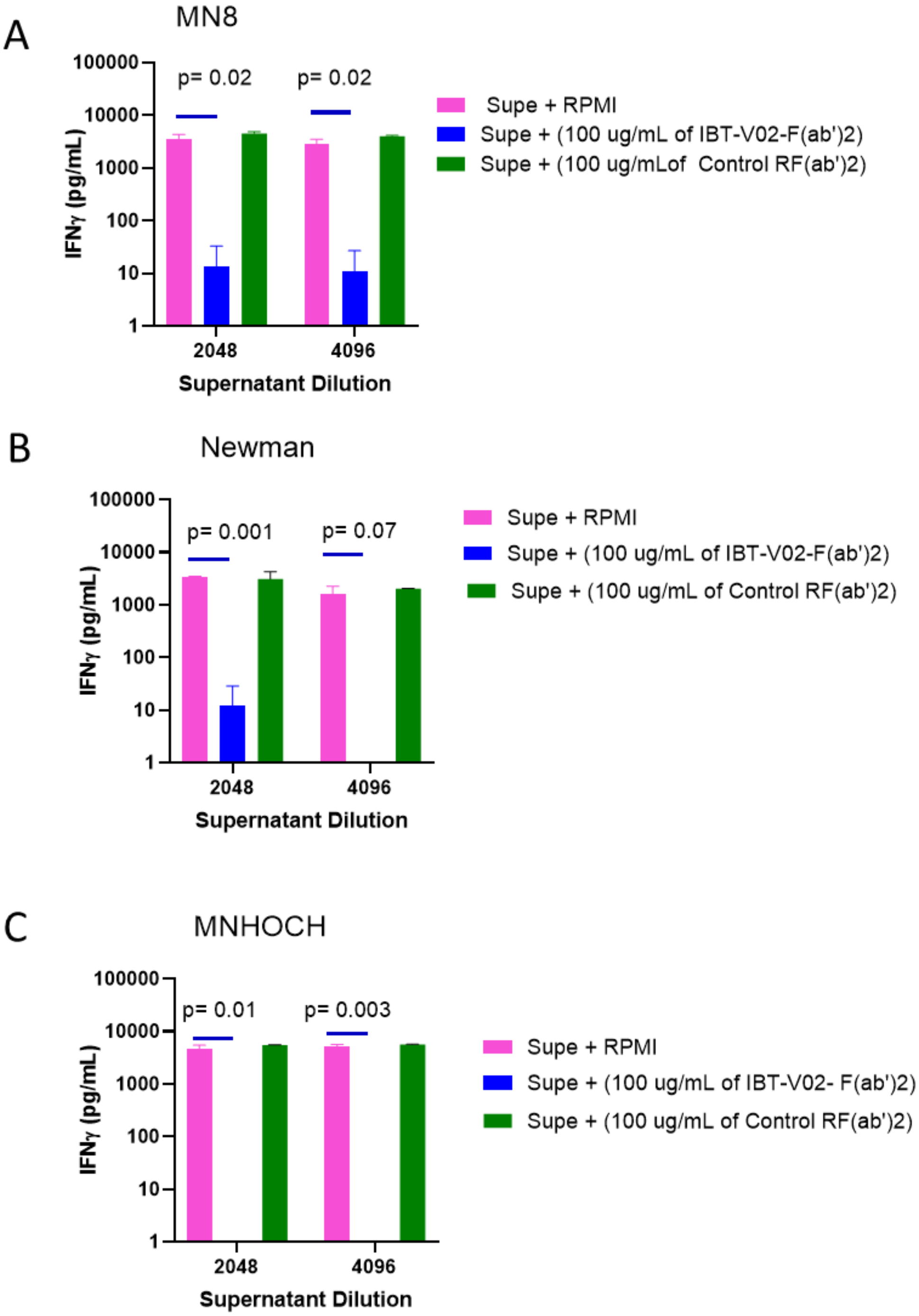

Supplementary Figure 1. Supernatant neutralization of sAgs by IBT-V02-F(ab')2 in human PBMC based assay. Supernatant (1: 2,048 and 4,096) dilutions were incubated either with 100 µg/ml of concentrations of IBT-V02-F(ab')2 or Control RF(ab')2. IFNγ level was measured by ELISA as a readout for the level of toxicity in different culture supernatants: **A)** TSST-1 producing strain MN8 **B)**

SEA producing strain Newman, and **C)** SEB producing strain MNHOCH. Data were analyzed by multiple t-test (Holm-Sidak method).

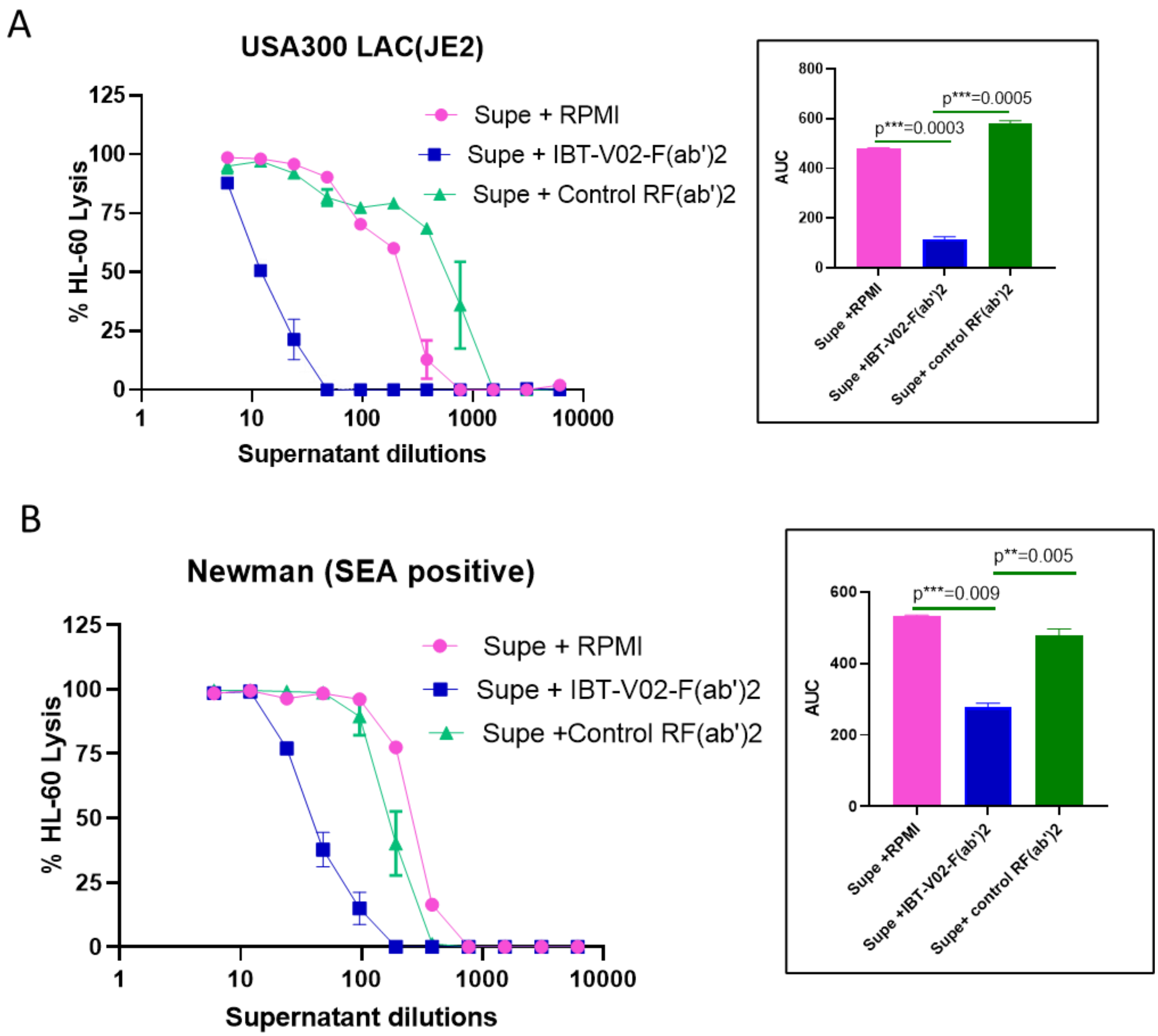

Supplementary Figure 2. Supernatant neutralization for leukotoxins by IBT-V02-F(ab')2 in HL-60 based assays. A) JE2 (USA300 LAC) and **B)** Newman. The area under the curve (AUC) is

shown in the inset in each plot. The significances were tested using Graph prism 8.4.2 by Two tailed unpaired t-test.

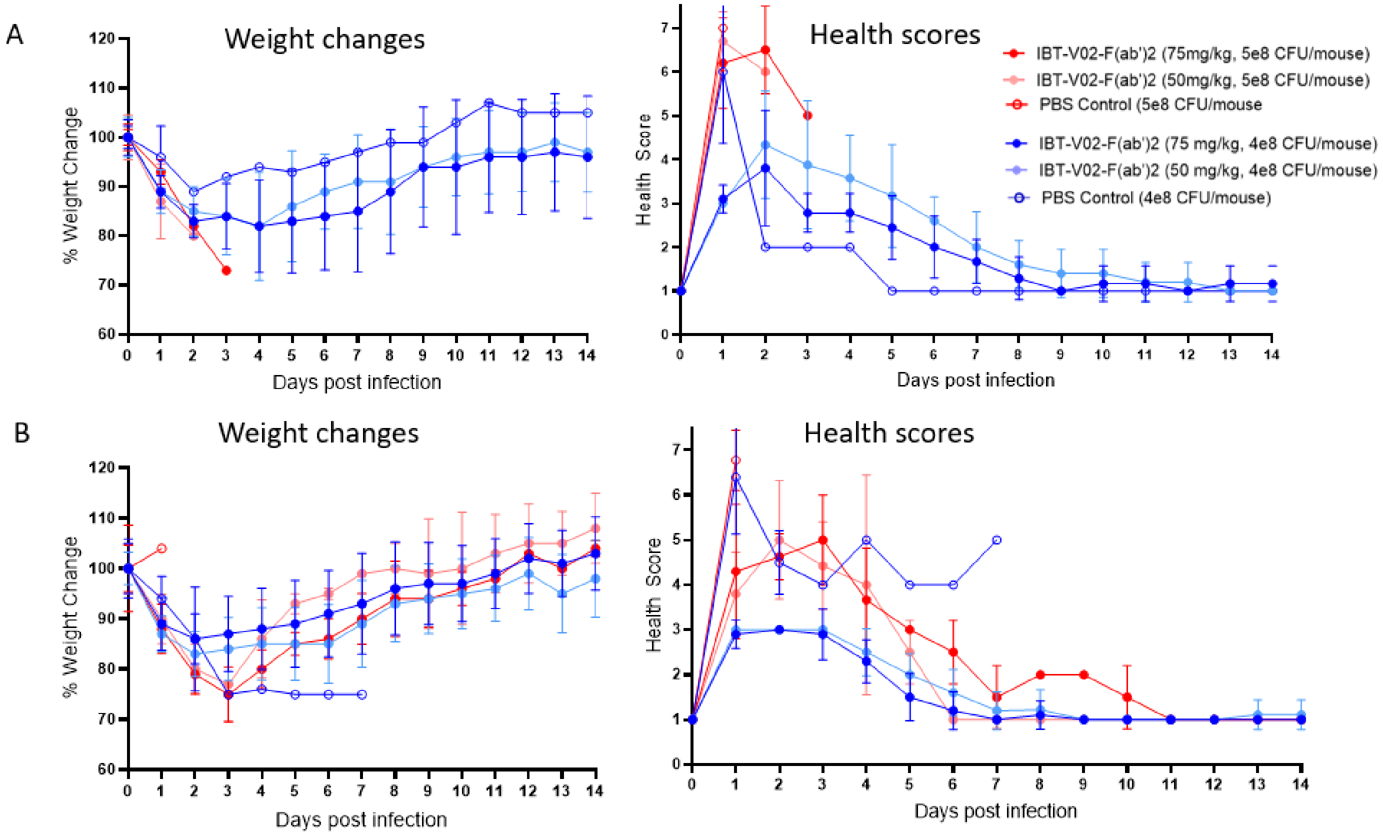

Supplementary Figure 3. Weight changes and health score monitoring. A) for bacteremia study as shown in Figure 9A and **B)** for bacteremia study as shown in Figure 9B.

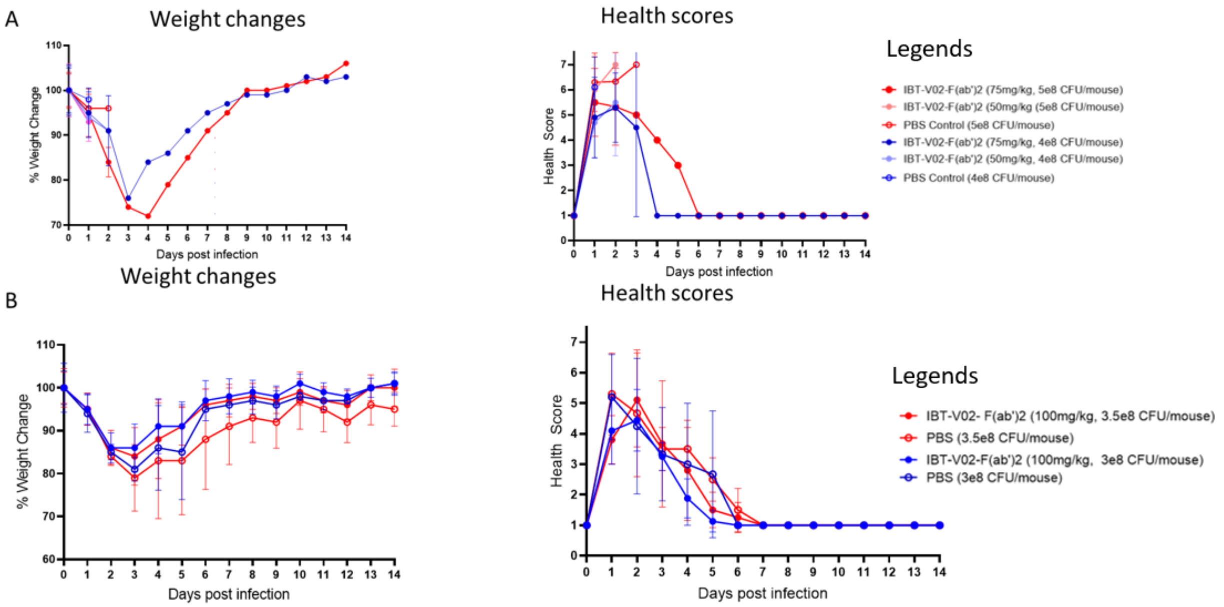

Supplementary Figure 4. Weight changes and health score monitoring. A) for pneumonia study as shown in Figure 10A and **B)** for pneumonia study as shown in Figure 10B.

Supplementary Table 1: Luminex binding titer of IBT-V02-F(ab')2, IBT-V02-IgG in comparison to negative control (non-immunized Rabbit F(ab')2 (RF(ab')2). Values are expressed as 50% effective concentration (EC_{50}, ng/ml).

Purified antibodies	Hla	LukS-PV	LukF-PV	SEA	SEB	TSST-1	LukAB
IBT-V02-F(ab')2	60	129	200	692	476	127	148
IBT-V02-IgG	69	130	234	663	445	151	169
Control RF(ab')2	6,585	1,648	5,103	>59,726	22,240	>62,544	990

Supplementary Table 2: Neutralization potency, 50% neutralization concentration (NC_{50} µg/ml) of IBT-V02-F(ab')2, IBT-V02-IgG and Control RF(ab')2.

Purified antibodies	Hla	Leukotoxins	Superantigens

		LukAB	PVL	TSST-1	SEB	SEA
IBT-V02-F(ab')2	22.70	4.72	23.12	1.66	0.37	16.73
IBT-V02-IgG	28.10	6.32	29.39	2.38	0.41	14.56
Control RF(ab')2	>556.00	49.60	>880.50	>278.00	>278.0	>278.0

Supplementary Table 3. Leukotoxins cross-neutralization potency (NC_{50} µg/ml) of IBT-V02-F(ab’)2, IBT-V02-IgG and RF(ab’)2

Purified antibodies	Leukotoxins Cross-neutralization		
	HlgAB	HlgCB	LukED
IBT-V02-F(ab')2	495.24	875.68	290.06
IBT-V02-IgG	2100.25	1,318.45	343.21
Control RF(ab')2	989.77	1,079.20	417.24
